# Rural and Urban Difference in Longitudinal Trends in Prevalence of Dementia in Medicare Claims and Survey Data

**DOI:** 10.1093/geroni/igab046.063

**Published:** 2021-12-17

**Authors:** Nasim B Ferdows

**Affiliations:** University of Oklahoma Health Sciences Center, Oklahoma City, Oklahoma, United States

## Abstract

Shortage of physicians in rural areas can lead to lower diagnosis and underestimation of dementia prevalence in these communities. We used data from the nationally representative Health and Retirement Study and a 20-percent sample of Medicare claims to study rural-urban differences in dementia prevalence. The survey dementia diagnosis is free from medical assessment while the claims diagnosis needs a physician diagnosis. We estimated the trends in dementia prevalence from (2002-2016) based on cognitive tests (using survey data) and diagnosis codes (using claims data) utilizing ordinary least squares regression. Dementia prevalence based on diagnosis codes declined in both urban and rural areas over the course of the study, with a sharper decline in urban areas. Dementia prevalence using diagnosis codes showed significantly higher rates in urban areas during all years (0.024 vs 0.018 in 2002 and 0.017 vs 0.013 in 2014 in rural vs urban areas, respectively). Dementia in the cognitive test sample was higher in rural areas (0.11 vs 0.08 in 2000 and 0.08 vs 0.7 in 2014 in rural vs urban areas), a difference that was significant only in 2004. Our results indicate lower dementia prevalence rates in rural areas in claims based sample compared to survey sample which its dementia prevalence is free medical assessment. Claims data are valuable sources for tracking dementia in the US population, however they are based on medical diagnosis.In rural areas, where there is shortage of physicians and a lack of access to health care services, claims based studies may underestimate dementia rates.

